# Supplementary material for: A Global Screen for Assembly State Changes of the Mitotic Proteome by SEC-SWATH-MS
Source: Cell Syst. 2020 Feb 26;10(2):133–155.e6. doi: 10.1016/j.cels.2020.01.001 (PMC7042714; doi:10.1016/j.cels.2020.01.001)

Q9BYB4 | GNB1L_HUMAN | GNB1L GY2 KIAA1645 WDR14 FKSG1
Monomer MW [kDa]: 35.618 Monomer expected elution fraction: 49

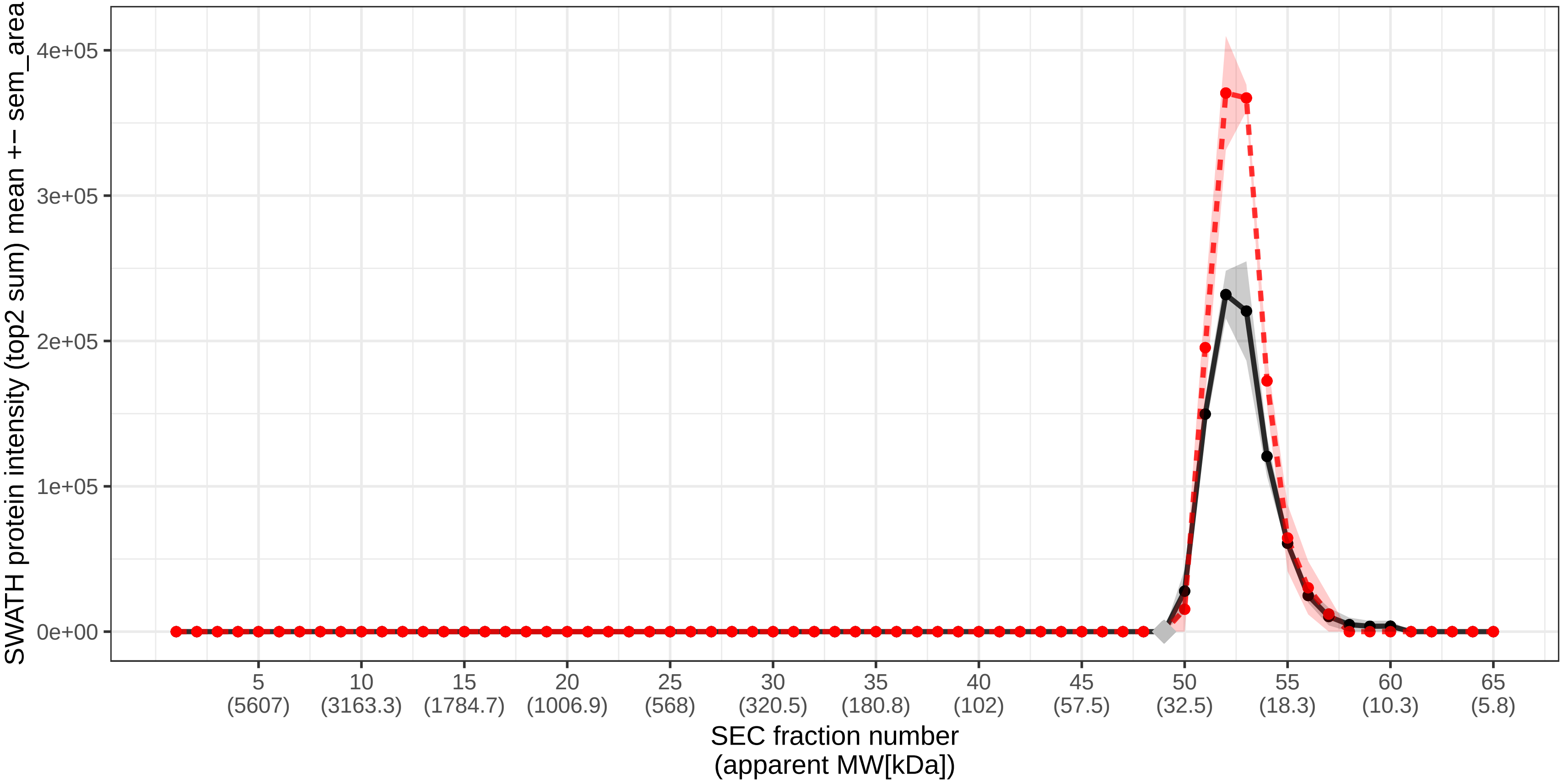

Supplement: Data S1. SEC-SWATH-MS Protein Chromatograms, Related to Figure 1 [file mmc6.zip › SECchrom_Q9BYB4_GNB1L_HUMAN_GNB1L_GY2_KIAA1645_WDR14_FKSG1.pdf]
